# Patients’ perspectives on factors facilitating adherence to tuberculosis treatment in Iquitos, Peru: a qualitative study

**DOI:** 10.1186/s12913-021-06329-z

**Published:** 2021-04-14

**Authors:** James Anthoney, Gilles De Wildt, Graciela Meza, John Skelton, Ian Newell

**Affiliations:** 1 College of Medical and Dental Sciences, University of Birmingham, Birmingham, UK; 2 Institute of Clinical Sciences, University of Birmingham, Birmingham, UK; 3 Facultad de Medicina Humana, Universidad Nacional de la Amazonia Peruana, Iquitos, Peru

**Keywords:** Tuberculosis, Adherence, Compliance, Peru, Qualitative, Adult, Therapeutics

## Abstract

**Background:**

Tuberculosis is a major global health problem and one of the greatest barriers to its control is poor adherence to treatment. Peru has one of the highest burdens of TB in South America, with an incidence rate of 123 per 100,000 populations. There is currently a lack of evidence in South America about factors that facilitate adherence to treatment, with most previous research focusing on factors that negatively influence adherence to TB treatment.

**Setting:**

This study was conducted in Iquitos, the capital city of the Loreto region, north-eastern Peru. Loreto has a high incidence of tuberculosis, estimated at 99 per 100,000 population, and a high poverty rate.

**Methods:**

Twenty face-to-face, semi-structured interviews were conducted at two healthcare centres. Data collected from the interviews was analysed using thematic content analysis.

**Results:**

Three main themes emerged from the data set. *Personal Qualities*, such as responsibility and determination, were perceived as important factors facilitating adherence. Participants described their *Trust in Healthcare Providers* positively, particularly focusing on their trust in clinical staff, although knowledge of tuberculosis and its treatment was limited. *Social Support*, from a variety of sources, was also seen as a driving factor for continued adherence.

**Conclusions:**

The results suggest that more emphasis should be placed on educating tuberculosis patients about their disease and its treatment. Additionally, consideration should be given to improving the social support available to patients, for example with tuberculosis support groups involving ‘expert’ tuberculosis patients.

**Supplementary Information:**

The online version contains supplementary material available at 10.1186/s12913-021-06329-z.

## Background

The World Health Organization (WHO) describes tuberculosis (TB) as a major global health problem and estimated that in 2018 there were 10.0 million new TB cases and 1.45 million TB deaths [1]. Peru has the highest incidence of TB in South America with a rate of 123 per 100,000 population [1]. Although Peru was removed from the WHO’s TB High Burden Countries list in 2002 [2], it has one of the highest rates of multi-drug resistant TB and extremely drug resistant TB in South America [1] and was added to the WHO’s High Burden Countries list for multi-drug resistant TB in 2005 [2].

Directly observed treatment short-course (DOTS), the WHO globally recommended TB treatment strategy [3], was implemented in Peru in 1990, providing the first evidence that widespread use of DOTS prevents new cases of TB [4]. The current DOTS treatment regime in Peru is outlined in Table 1. Prior to the implementation of DOTS, only 50% of patients in Peru diagnosed with TB received the full course of treatment, resulting in high rates of drug resistant TB [5]. Although DOTS has been effective in both preventing and treating TB [6], with 86% of new Peruvian cases treated in 2017 [1], poor adherence to TB treatment, in the form of either refusing to start the treatment or failing to complete the full course, is still a major barrier to control of the disease [7]. Non-adherence is a complex issue with many causes, such as prolonged treatment duration, high numbers of pills, being observed and having to attend clinics daily for medication [8]. It also has serious consequences, including drug resistance, prolonged infectiousness, relapse and death [8]. The WHO defines adherence to long-term therapy as “the extent to which a person’s behaviour … corresponds with agreed recommendations from a health care provider” [7].

**Table 1:** Peruvian dots treatment regime

Scheme 1	Scheme 2
**Indicated for:**	**Indicated for:**
Never-treated patients	Relapses and those who had previously discontinued treatment, then returned
**Medications:**	**Medications:**
Isoniazid (H), Rifampicin (R), Pyrazinamide (Z) and Ethambutol (E) - HRZE	Rifampicin (R), Isoniazid (H), Pyrazinamide (Z), Ethambutol (E) and Streptomycin (S) - RHZES
**Duration:**	**Duration:**
6 months, 82 doses	8 months, 125 doses
**Phase 1:**	**Phase 1:**
50 doses (daily from Monday to Saturday) of HRZE	75 doses (daily from Monday to Saturday) of RHZES then one month (daily Monday to Saturday) of RHZE
**Phase 2:**	**Phase 2:**
32 doses (twice a week) of RH	40 doses (twice a week) of RHE

A literature search was conducted using Medline and the Cochrane Library, which returned two relevant results to this study. Culqui et al. quantitatively examined non-completion of TB treatment in Peru, finding that non-adherence was associated with male sex, previous non-compliance, malaise during treatment, illegal drug use and poverty [5]. However, the themes surrounding adherence are intricately linked and likely to have a combined effect [9], making it difficult to quantitatively measure factors that independently lead to non-adherence. Therefore, qualitative research may be more appropriate help us understand patient experiences and the barriers to, and facilitators of, treatment adherence, which are needed to improve treatment outcomes [10].

Munro et al. conducted a systematic review finding two relevant qualitative studies in Latin America [9]. They found eight major factors across the global literature that influenced adherence, including interpretations of illness and wellness, financial burden of TB treatment and knowledge, attitudes and beliefs about TB treatment, which led them to conclude adherence to TB treatment has a complex and dynamic nature [9]. Furthermore, Munro et al. emphasised that more research is needed to help understand people’s experience of TB and its treatment, which will improve treatment adherence by allowing the development of more patient-centred approaches [9].

Despite both qualitative and quantitative research examining TB treatment adherence being readily available [5, 9, 11], previous research has been primarily focused on barriers to adherence. This has resulted in a gap in the evidence regarding the factors that facilitate adherence. Furthermore, the regional government of Loreto, the region of Peru in which this study was conducted, identified the factors associated with acceptance of TB treatment and poor adherence as being a health research priority [12]. The objective of this study is to investigate the factors that facilitate adherence, as well as the barriers, to curative TB treatment from the perspective of TB patients in Iquitos, the capital city of the Loreto region of Peru. This study hopes to achieve this aim by exploring the personal, social, structural and health service factors of participants in detail.

## Methods

### Aim

To investigate the facilitating factors and barriers to adherence to curative tuberculosis treatment from the perspective of patients in Iquitos, Peru. To achieve this aim, the personal, social, structural and health service factors participants perceived to be important to their treatment adherence were explored in detail.

### Design

Face-to-face, semi-structured interviews were conducted that lasted between 20 and 40 min between January and February 2017. Interviews were selected as the most appropriate qualitative method of research because they allow for in-depth exploration of participants’ perceptions and beliefs. The semi-structured nature of the interviews allowed the principal researcher to find a balance between having minimal control of the conversation [13] and flexibly directing the interview’s focus [14]. Each interview was based on a topic guide (Additional file 2) and consisted mainly of open-ended questions, which allowed participants to give in-depth answers and influence the direction of the interview, to some extent, towards the factors that they perceived to be important. With this focus on perceptions in mind, not all participants were explicitly asked about known risk factors, but their views on these factors were explored if they came up. The topic guide was developed with reference to Munro et al. [9] and Culqui et al. [5]. A constant comparison approach was taken using the English translation of the data, thereby allowing the topic guide to be adapted during the process, to allow for the generation of theories that are more integrated, consistent and plausible [15]. The endpoint of data collection was reached when it was deemed analytical saturation had occurred, based upon when no further themes were emerging. No repeat interviews were conducted.

### Setting

This study was conducted in Iquitos, the capital city of the Loreto region, north-eastern Peru. Loreto has one of the highest incidence rates of TB in Peru [16] and a high poverty rate [17], which has been recognised as influencing adherence in other settings [5, 9]. Loreto also has a lower education level than some other areas of Peru, with 39.7% of males and 43.2% of females not completing primary education, compared to 21.7 and 25.3% respectively in Lima [18]. Recruitment and data collection were conducted in the Centro de Salud San Juan, in the San Juan Bautista district, and the Centro de Salud Moronacocha, in the El Centro district. Both of these centres follow the DOTS strategy for TB, through which patients are required to attend daily to collect their medication, with the record of their attendance used to monitor adherence. All participants had been adherent to their current course of treatment based on their daily attendance records, although some did explain that they had failed to adhere to previous courses. Individuals who participated in the study came from all walks of life, for example, taxi drivers, musicians and students.

### Sampling

Convenience sampling of TB patients attending either healthcare clinic was used, based upon the inclusion and exclusion criteria outlined in Table 2. Clinical staff were used to assist the principal researcher in determining which patients fit the inclusion and exclusion criteria. Convenience sampling was employed due to time and resource constraints and the low numbers of TB patients in total at the healthcare centres. Initially, purposive sampling to achieve an equal gender split was attempted but had to be stopped due to the limited number of female TB patients at the healthcare centres. All participants were approached in person and given a Patient Information Sheet outlining the aims of the research and relevant information about the researcher. Participants were also given the chance to ask the researcher questions and informed consent was given by all. Three TB patients who were approached declined to participate, stating time constraints as the reason. It was not known whether these patients differed to the participant group in relevant characteristics.

**Table 2:** Inclusion and exclusion criteria

Inclusion Criteria	Exclusion Criteria
Male or female patient currently being treated for TB at either San Juan or Moronacocha healthcare centres	Any patient unable to give written or verbal informed consent
Legal adult in Peru	Any patient the clinician advises is too unwell to participate for the whole interview
Lives in the Loreto region	Not Spanish or English speaking

### Data collection

All interviews were conducted in Spanish, with the aid of a translator, who was briefed on the purpose of the study, ethical issues, TB-specific terminology and interpretive practices in interviewing [19] before data collection began. Interviews were audio-recorded and transcribed verbatim. All transcripts were anonymised to ensure confidentiality and encrypted using 128-bit encryption. All data was stored on the secure University of Birmingham server. Transcription occurred concurrently with data collection to allow for the use of a constant comparison approach. All participants were free to withdraw from the study up to 2 days after they were interviewed. There was no reimbursement for participation in the study. A random sample of 10% of the interviews were transcribed in Spanish and translated separately as a quality check. These transcripts were checked against the transcripts produced by the principal researcher.

### Analysis

Thematic analysis was used because it allows for richly detailed description and interpretation of the data [20]. Braun and Clarke’s six-phased approach to thematic analysis was carefully followed in a systematic fashion to manually analyse the data [20]. No deviations were made from Braun and Clarke’s approach during the analysis. Munro et al.’s Model of Factors Affecting Adherence was initially used as a theoretical framework [9] because of its utilisation in the production of the topic guide. However, the themes that emerged from the data did not adequately fit this framework so an inductive, data-driven form of analysis was taken. As a result, the themes produced are not intended to supplement an existing theoretical framework but stand as themes in their own right. External validity was improved using analytical triangulation. An additional researcher, was concurrently working on another qualitative study in Iquitos, completed phases one and two of Braun and Clarke’s approach for a random sample of 25% of the data. The congruence of the initial codes produced by the principal researcher and additional researcher were checked against one another to circumvent any personal biases of the principal researcher [21]. Respondent validation of transcripts or findings was not used due to time and resource constraints and the feasibility of contacting and communicating with participants after they had been interviewed.

## Results

Data saturation was reached after 20 interviews. Participants were equally distributed by age group (above or below 30 years of age) with a median age of 31.5 years (interquartile range = 25–67 years). Six females and 14 males participated. The participants were at varying stages of their TB treatment courses, ranging from Day 1 of treatment to only having 2 days left of treatment. The median number of days of treatment was 90 (interquartile range = 24–135 days). Educational status was grouped into ‘Received or currently in secondary or university education’ (*n* = 12), ‘Received some level of primary education’ (*n* = 5) and ‘Never received education’ (*n* = 3). Occupation was the only socioeconomic data recorded and varied widely between participants. The largest occupation groups were students (*n* = 5), manual labourers (*n* = 4), market sellers (*n* = 3) and unemployed (*n* = 3). All the participants were local residents. No discernible pattern was found between these demographic factors for the themes that emerged. The main themes that emerged were personal qualities, trust in healthcare providers, health beliefs and social support.

### Personal qualities

A strong sense of determination to finish the treatment and the importance of personal goals were apparent in many of the interviews. In these cases, motivation was one of the most important factors in the participant’s continued adherence.

*I try my best every day to take the pills and that makes me feel so happy … I am determined to finish my treatment. [Participant 01 – Male]*

Although the specifics of the personal goals varied, they often centred around a return to normality. The motivation to achieve their goal was often instrumental to their hopes of treatment completion, in what many saw as a potentially difficult journey through the TB treatment process.

*My aim is to feel better and to be cured. I want to be able to do normal things again. [Participant 06 - Male]*

*I must finish the treatment for the sake of my baby. I didn’t finish the treatment the first time I had tuberculosis but this time I am determined to finish it for my family. [Participant 04 - Female]*

A few participants implied that a person’s character determined whether they possessed the motivation needed to adhere to the treatment. The word ‘worthwhile’ in the following quote is interesting linguistically and an example of how using a translator can impact qualitative data. While the participant may have indeed meant that a person with ‘worth’ is more likely to finish the treatment, it should also be considered that this could have also been translated as ‘a person with values’. This would fit better with the sentiment presented by many participants that patients have a moral obligation, or responsibility, to finish treatment, implying that those with moral values would find adherence easier.

*Whether a person finishes their treatment also depends on their character … If you are a worthwhile or strong person you will finish the treatment. [Participant 20 - Male]*

Responsibility was often closely associated with personal motivation and agency in relation to finishing the treatment.

*You have to approach treatment of this disease with responsibility. People have to finish the treatment if they want to be cured. [Participant 12 - Male]*

When discussing the idea of default, either in the context of others or themselves, the attitude of many participants was that non-adherence and irresponsibility were practically synonymous.

*The people who stop taking their treatment are irresponsible because they don’t care about their health. [Participant 03 – Male]*

*The only reason that would cause me to quit treatment was if I was irresponsible … I would call myself irresponsible if I left the treatment because the people that come here have the idea in their mind that they must finish the treatment and after that they will be cured. [Participant 13 - Male]*

‘Responsible’ was a word that participants were quick to assign to themselves and keen to dissociate from people they perceived had negatively impacted their health or treatment adherence. Responsibility appeared to refer to being responsible towards oneself more than towards one’s family or healthcare professionals. This often stemmed from a fear of not being able to work or study if the illness was not treated. For some participants, responsibility extended past being a solely personal matter into becoming an inspiration to help other TB patients.

*I want to help other people with the disease. [Participant 01 - Male]*

*I would like to be an example that other people could follow. [Participant 19 - Male]*

### Trust in Healthcare Providers and Health Beliefs

While participants valued personal qualities as important drivers of adherence, they also recognised the importance of external facilitators. Most patients’ perceptions of the organisation of their healthcare were positive, whilst negative attitudes were rare. Good organisation was often closely associated with patients’ treatment by staff.

*My treatment has been well organised here … the people here treat me very well, so I am very happy. The nurse treats me so well, she helps me a lot, which is good for me. [Participant 15 - Male]*

Linked to the perception of good staff treatment was participants’ trust in the clinical staff. With very few exceptions, participants had complete trust in their doctor regarding all aspects of the disease and treatment. While responses such as this could be considered a result of social desirability bias, participants who gave responses with this sentiment did appear to genuinely believe they were following their doctor’s guidance accurately.

*I do everything the doctor says. [Participant 01 - Male]*

*If the doctors tell me I am going to have treatment for this long I will, because the doctors know. [Participant 03 - Male]*

This unwavering trust resulted in a strong sentiment that if the doctor’s advice was followed, their TB would be cured.

*I am going to finish the treatment because the doctor told me to. He told me to take the pills for 6 months. [Participant 12 - Male]*

While trust in clinical staff was perceived to be a facilitator of adherence, it may also be a contributing factor to the significant barrier of limited patient knowledge. In this context, there is a strong link between trust in healthcare providers and patients’ health beliefs. Participants perceived their care to be controlled by their doctor, rather than themselves, and, as such, educating themselves about the disease or treatment held limited value.

*I don’t know anything about the treatment or how long it is. [Participant 10 - Male]*

Similarly, there was often confusion about exactly when and how cure was determined. Some believed they would be cured when they felt better, some thought it was dependent on finishing the treatment and others saw diagnostic tests as the determinant of cure. There was also limited knowledge regarding the disease itself. Very few participants identified TB as a bacterial infection, with some believing it was viral and many relating a complete lack of knowledge pertaining to the natural history of TB. In some cases, participants were not only lacked knowledge of the disease but also possessed potentially damaging conceptions about the disease. The following quote illustrates an interesting conceptualisation of TB in which the disease could be physically removed from the body.

*I don’t understand what tuberculosis is … I don’t know why I have this pain in my lungs. Sometimes I try to beat the pain out of my lungs. [Participant 17 – Female]*

Participants’ health beliefs regarding TB varied widely, especially in respect to disease contraction. Although some participants correctly identified coughing and sneezing as the method of transmission, many relayed the false ideas of transmission through contagious cutlery/crockery and talking or living with an infected person. Alimentation was perceived as the most important determinant of infection. When asked how people become infected with TB, Participant 19 suggested that a person’s diet determines whether they get infected.

*When they don’t eat very well … It depends on the food that people eat, the food needs to have vitamins. [Participant 19 - Male]*

*It depends on food... I was told that I got tuberculosis because I was not eating very well [Participant 18 – Male]*

The origin of this health belief probably stems from the concept that active TB infection can arise in people with the latent form of the disease when they have a weakened immune system. Good nutrition is linked with good health and so it seems logical that healthcare professionals and lay people would advise an ill person to improve their diet either as a general health benefit or to directly improve immune strength.

### Social support

External facilitators of adherence extended beyond clinical healthcare to include social support. By and large, the most significant social support participants received was from their family. For many, the support of their family, both emotional and financial, was invaluable to their continued adherence to the treatment regimen.

*It was so difficult to continue working but thankfully my husband helps me a lot. He doesn’t want to leave me, which I am very grateful for, so I still live with him … My family helps me a lot … They are very supportive. [Participant 07 - Female]*

Most responses regarding family support implied a strong sense of inherent trust between the participant and their supportive family members. As such, it is unsurprising that advice from family members was also considered important by some participants, and was often intertwined with the participants own personal motivations.

*My father and my husband tell me to take care of myself and to take care of the baby. [Participant 04 - Female]*

In cases where participants lacked family support (*n* = 3), they often portrayed a feeling of isolation and indicated that they might consequently struggle with aspects of their health or treatment.

*The first time I told my family, they didn’t want to come near me. It was horrible for me. They treated me so badly because they wanted me to move out and be far away. I was so angry. I didn’t want to be treated like this by my family. [Participant 17 - Female]*

*I am living alone and I struggle to eat in the right way. [Participant 02 - Female]*

When participants who lacked family support were asked if they believed this would affect their likelihood of adherence, they usually suggested that it would not. Interestingly, these participants also seemed to be the most likely to be non-adherent due to other factors, such as illegal drug use.

Perceptions of community support were mixed. While most participants related a fairly neutral opinion towards the community impact on their adherence, none stated that their community had a greater influence than their family, and a few believed the community should never be given the chance to affect one’s adherence.

*None of the people in my neighbourhood know … I preferred it to be a kind of secret. [Participant 01 - Male]*

*If you have a problem, it should be kept inside your house. Your neighbours should not know about it. [Participant 03 - Male]*

Positive community influence was generally viewed as being limited. However, for every participant who knew another TB patient, their relationship with this person was perceived as beneficial, and their opinion of community support was more positive.

*I first asked for advice from my family and then other people who I know who had tuberculosis … the advice they give me helps a lot. [Participant 13 - Male]*

*I also have a neighbour who lives opposite me who has tuberculosis as well … she had it first so she helped me a lot. [Participant 16 - Female]*

## Discussion

Participants described both internal and external factors they believed were important in facilitating their TB treatment adherence. The internal factors were focused on positive personal qualities, such as determination, strength of character and responsibility. The significant external factors included well-organised healthcare and social support, which consisted mainly of family and fellow TB sufferers.

It is important to appreciate the emphasis placed on patients’ perspectives. This was intentionally done in recognition of Munro et al.’s recommendation that more research is needed to understand patients’ experiences of TB treatment, particularly in the context of lay conceptualisation of illness and wellness [9]. Furthermore, Vermeire et al. found in their review of compliance that the most salient influences on adherence are patients’ general beliefs about medicine and specific beliefs about the treatment [10]. The aim of the study was to highlight the factors that TB patients in this setting perceive to be important regarding their adherence to treatment.

Munro et al. advised caution when attributing adherence to personal qualities and motivation because they can cloud the importance of other influences [9]. However, participants perceived an individual’s character and their worth to be important facilitators of adherence. The reason that participants focused on personal qualities as important drivers of adherence could be seen as an effort to achieve a level of autonomy regarding their treatment. Characteristics such as determination and responsibility are qualities that participants believe they already possess, or can at least work towards. Adherence then becomes the demonstration that they have attained these qualities. Many participants were eager to convey that they thought of themselves as worthwhile and responsible. This may be in contrast to an understanding of the disease or treatment, which participants perceived as beyond reach. Very few participants confidently provided an understanding of their condition or showed evidence of trying to improve their knowledge. By making attainable factors the central determinants of adherence and relegating seemingly unattainable factors to the concern of healthcare professionals, participants may feel they can retain the locus of control for their treatment. This is particularly pertinent in this setting, where education levels are low, and the DOTS regime can be seen to reduce patients’ autonomy.

Furthermore, participants were quick to dismiss those who defaulted as irresponsible, implying that non-adherence should have some accountability attached. When pressed on why default was irresponsible, participants often suggested that one must hold themselves accountable for their own health, thereby attributing adherence once again to an internal, controllable factor. This reiterates the assertion that adherence is determined, not by uncontrollable factors, but by personal qualities, which is within the control of patients.

The rejection of any idea of justified non-adherence may stem from a paternalistic doctor-patient relationship that was implied by many participants. The absolute trust in healthcare professionals exhibited by many of the participants, combined with their limited knowledge of the disease and its treatment, results in an imbalanced power relationship between the doctor and patient. The impact this paternalistic relationship has on adherence is complex. On the one hand, Vermeire et al. and Munro et al. suggest that adherence is improved when doctors are considered expert advisors, rather than sole decision makers, and patients’ autonomy and self-determination are acknowledged [9, 10]. On the other hand, in a setting such as this, where lack of knowledge among patients could be a major barrier to adherence [10], the chances of default may be greatly reduced when patients religiously follow their doctor’s advice. If the latter argument is to be followed in this setting, the implication would be to maintain and improve the current high standards of healthcare provision to TB patients to further improve patients’ trust in healthcare providers.

It is generally accepted in other studies that lack of education is a barrier to adherence. McNally et al., in a qualitative study in Iquitos, found that high patient knowledge is an important factor in positive patient outcomes in the context of multi-drug resistant TB, providing evidence for this theory specific to the setting of Iquitos [22]. Although this study did not find lack of understanding to be a significant barrier to adherence, this is likely to be due to only adherent patients being interviewed. The multiple erroneous health beliefs presented show that significant consideration must be given to the way in which clinicians communicate ideas to newly diagnosed TB patients and the cultural implications of their words. Finding the appropriate level of information to provide may prove difficult because of the varying levels of education amongst patients in this setting. However, if a comprehensive approach to improving patient education that is both culturally and informationally appropriate is taken, then patient autonomy and agency could be improved.

Another important facilitator of adherence was a strong sense of family support. For many of the participants, their health beliefs were strongly influenced by their family. Further to the emphasis on greater patient education, consideration should also be given to encouragement of greater family involvement in the treatment process, including education of family members about the disease and treatment. The benefits of this proposal could include even more significant positive family support and reducing the likelihood of conflicting advice from the patient’s doctor and their family, which participants found hard to reconcile.

Knowing another TB patient was also seen as a factor that facilitated adherence. Given their common burden, other TB patients were able to offer a form of support that family members could not provide. When coupled with the desire expressed by some participants to help and be an example to others with TB, this presents an opportunity to improve the social support TB patients are offered in this setting. The results of this study suggest that consideration should be given to TB support groups with input from ‘expert’ TB patients, although, it should be recognised that TB patients already have a significant time cost associated with their treatment due to the DOTS regime. This type of support could be particularly helpful for those who lack family or other social support.

The main implications for practice are that patients perceive themselves as likely to succeed because they are people of worth and character. This suggests that healthcare professionals should be trained to recognise and build on this perception, particularly in the low education and DOTS regime setting of Iquitos, Peru. Similarly, participants speak of the value of family support. Healthcare professionals should be supported, therefore, in recognising the role of the family in the patient’s care and building on this.

### Limitations

As a qualitative study, the results are not generalizable to other settings or populations. However, they do give an insight into this setting and aim to further our understanding of TB patients’ perspectives on the disease and its treatment. The use of a foreign researcher may have also had a cultural impact on the data, either through the way participants answered questions or how the answers were interpreted. Furthermore, the participants may have been influenced by social desirability bias given that the interviewer had an association with the healthcare centres. The interviewer tried to reduce this through their explanation of confidentiality and almost all participants seemed to be giving open and honest answers and showed a genuine interest in the research.

Using a translator whilst interviewing is a limitation because of how it affects the flow, especially when terminology or concepts don’t translate exactly and have to be explained, although, Twinn found no significant differences in the major categories generated when using translators [23]. Furthermore, the use of a translator may have impacted the interviewer-interviewee relationship. It did not appear to result in an asymmetrical power relationship because both interviewer and interviewee may have felt unsettled when they couldn’t understand the conversation.

One aspect considered of potential relevance at the outset was the possibility of variation by gender, a recommendation for further research offered by Munro et al. [9]. In the event, the number of females who consented was too small to reach clear conclusions. All that can be stated is that, within this small data set, there was no evident difference, shown by variation in gender of the quotations used. Achieving a representative sample of participants based on other factors is also difficult using convenience sampling but it was felt that the other factors documented, including age, education and occupation were representative within the limitations of a small sample size.

Perhaps the most significant limitation of this study was that only patients who were adherent to TB treatment were interviewed, thereby not taking the views of non-adherent TB patients into account. It is clear that these views are important when exploring adherence, as shown by other studies. However, the use of only adherent patients has allowed this study to more easily focus on factors facilitating adherence to address the gap in the literature, as well as the barriers.

### Further research

If greater education is to be a significant facilitator of adherence, more research into effective behavioural change in TB treatment adherence is needed. Valente et al. suggests that the way behavioural change occurs in a population and the behavioural model it follows depends on the nature of the behaviour, the existing level of knowledge and the population demographic [24]. Without properly understanding these factors, attempting to increase patient knowledge of TB and its treatment may negatively impact adherence by creating dissatisfied patients. Therefore, if improving adherence through improving education is to be achieved, further research into the patient demographic and their knowledge, attitudes and practices is needed. With this knowledge, it would also be easier to facilitate greater patient autonomy and self-determination, thereby moving towards Munro et al.’s aim of more patient-centred approaches to TB treatment adherence [9]. It is clear that further research is needed to investigate any differences between gender. Additionally, there is currently a lack of qualitative research surrounding TB treatment adherence in South America, which needs to be addressed.

## Conclusions

This study identified several factors that facilitate adherence to TB treatment in Iquitos, including personal qualities, trust in healthcare providers, health beliefs and social support. The study also identified areas of care that could be considered in need of improvement. Patients’ limited knowledge about the disease and its treatment should be addressed and thought should be given to ways in which patients’ social support can be enhanced.

## Supplementary Information

**Additional file 1.** Coreq checklist.

**Additional file 2.** Topic guide.

## Data Availability

The datasets used and analysed during this study are available from the corresponding author on reasonable request.

## Abbreviations

DOTS: Directly Observed Therapy Short-Course
TB: Tuberculosis
WHO: World Health Organization
