# Supplementary material for: Patients’ perspectives on factors facilitating adherence to tuberculosis treatment in Iquitos, Peru: a qualitative study
Source: BMC Health Serv Res. 2021 Apr 14;21:345. doi: 10.1186/s12913-021-06329-z (PMC8048224; doi:10.1186/s12913-021-06329-z)
Supplement: Supplementary file 2 — Additional file 2. Topic guide. [file 12913_2021_6329_MOESM2_ESM.pdf]

APPENDIX 2 - TOPIC GUIDE

Introduction

Opening line:

Hello, my name is James Anthoney and I am a researcher at the University of Birmingham, in the UK.

Thank you very much for agreeing to participate in my study.

Interview information:

- Study purpose
  - The aim of this study is to explore tuberculosis patients' ideas and opinions of the factors that influence whether they complete their treatment
  - We hope that the results will help improve the understanding of healthcare workers in Loreto about tuberculosis treatment, which may lead to better care
- Interview content
  - There are many factors that influence whether patients complete their tuberculosis treatment and I will be asking you which factors you find important and why
- Duration
  - The interview will last up to one hour
- Interview process
  - The interview will be recorded on this phone and the recording will be securely stored
- Confidentiality
  - Everything you say in the interview will be kept completely in confidence by both myself and [*insert translator name*]

- Neither [*insert translator name*], nor myself, work for the healthcare centre and we will not be reporting your answers to the staff here
- All the information you give will be anonymised so even if what you say appears in the results of the paper, no-one apart from us will know you said it

- Questions
  - Do you have any questions at this point?

Consent:

You have already signed the consent form but can I ask you to confirm that you understand what the purpose of this study is and that you are happy to be interviewed?

And do you know that you can withdraw up from the study up until two days after this interview?

Demographic:

- How old are you?
- For the purpose of the recording, can you state whether you are male or female?
- What is your occupation?
- What age did you leave school?

Start the Topic Guide:

Thank you very much. Are you ready to start the interview questions about tuberculosis treatment now?

- Tell me about your TB
  - How long have you had it?

Topic	Questions and probes

**Treatment organisation and care**	How well organised do you think your treatment and care have been here?<br>• How easy is it for you to get here?<br>• Do you think this healthcare centre is well run?<br>• What do you think could be improved?<br>What reasons might stop you from coming for treatment?
**Financial burden of treatment**	What costs have been involved with your treatment?<br>• Have there been many unexpected costs?<br>How has the treatment affected your ability to work?<br>• Could you describe any fears or concerns about work that you've had since your diagnosis?<br>• Do you think that you had to make a choice between work and treatment?<br>How has the treatment affected the rest of your household?
**Community and household influences**	How have your household influenced you taking your treatment?<br>• What attitude have members of your household had to your disease and the treatment?<br>• Did you have any fears or concerns about telling members of your household about your diagnosis?<br>• To what extent has your role in the household affected you taking your medication?<br>• How well supported have you felt by your household?<br>What effect has your community had on you taking your medication?

	• Do you think that members of your community have treated or viewed you differently since you started treatment?<br>• What advice have you been given by members of the community or your household about the treatment?
**Treatment side effects**	What do you understand about the treatment side effects?<br>• If you have had any side effects, could you describe them?<br>What would you do if you experienced side effects?<br>• How do you think healthcare workers would react if you told them about the side effects?
**Beliefs about the disease**	What do you understand about tuberculosis as a disease?<br>• Do you know where tuberculosis comes from?<br>• How do people become infected with tuberculosis?<br>• What do you think causes some people to become infected and not others?<br>• How were your beliefs about the disease different from what the doctor told you when you were diagnosed?<br>• How have your beliefs about tuberculosis changed since you were diagnosed?
**Knowledge, attitudes and beliefs about TB treatment**	What do you understand about the treatment you are given for tuberculosis?<br>• What does the medication do?<br>• How effective do you think the treatment is?<br>Do you know how long you will be taking treatment for?<br>• Do you know why you have to take the treatment for so long?

	• How difficult do you think it will be to take treatment for that long?<br>How important do you think it is to finish the treatment?<br>What do you think about other medications, not from the doctor, that may be available?<br>• Would you consider using medicines from local healers?
**Conceptualisation of illness and wellness**	How do you think you know that you are cured?<br>• Would you think you were cured if you felt better but treatment hadn't finished yet? If so, would you continue treatment?<br>• How would feeling better affect your attitude towards treatment?<br>What would you think about the treatment if it made you feel worse?<br>• When would you consider stopping treatment if it made you feel worse?
**Personal characteristics**	How do you think your character has affected you taking your medication?<br>Do you think your opinion of the disease and treatment affects the likelihood of finishing the treatment regime?<br>How important do you think it is for you to be in control of your treatment?<br>• How does being observed taking treatment affect you?
**Gender differences**	How do you think men and women approach treatment differently?

Closing remarks:

Is there anything else that you think is important for me to know about tuberculosis treatment for you personally or for the people in Iquitos?

Thank the participant:

The interview has now finished. Thank you very much for your time. The information you have given is very important for this study. If you do have any questions about the study, please feel free to use the contact details on the information sheet. Thanks again.

Turn off the audio-recorder

This topic guide is more formal than the questions that will actually be used in the interviews. It provides a guide for the interviewer from which to direct the flow of the interview. The pilot interviews will be used to help the researcher gauge the level at which to pitch the questions.

Reflection

A short write-up immediately after the interview, covering:

- The main points made
- New or unexpected ideas/information
- How the interview went
    - Problems with the topic guide
    - Interpretation problems
- Whether data collection is nearing saturation

NB: This Topic Guide was changed during the interview process as a result of the constant comparison approach and discussion with the translator so the questions actually asked during interviews varied significantly.
